# 1118. Case Study. Juggling on a Tight Rope: Resuscitating a Patient with a Large Burn with Malnutrition

**DOI:** 10.1093/jbcr/irag033.292

**Published:** 2026-04-06

**Authors:** Molly Marsh, Felicia N Williams, Jamie L Hollowell, Alexandra R Coward, Booker King

**Affiliations:** North Carolina Jaycee Burn Center, Chapel Hill, NC; University of North Carolina,, Chapel Hill, NC; North Carolina Jaycee Burn Center, Chapel Hill, NC; University of North Carolina, Durham, NC; University of North Carolina, Durham, NC

## Abstract

**Patient Presentation (age range, injury details, relevant history):**

This patient is a 30-year-old male with a past medical history of alcohol use and substance use disorders and bulimia nervosa presented to our burn center with a 42% total body surface area mixed second and third-degree flame burns. He arrived at our center three hours post-injury; he underwent emergent escharotomies to his left lower extremity, and we initiated fluid resuscitation.

**Clinical Challenges:**

Fluid resuscitation and adequate nutrition are essential to survive and heal a large burn. Adequate but not excessive fluid resuscitation is a well-documented challenge, even more so in the setting of patients with baseline malnutrition or disordered eating. We present a case of a patient with a history of malnutrition prior to admission that exacerbated the complications of burn resuscitation. He also required escharotomies upon admission, which further increased fluid losses. Further complicating resuscitation, the patient refused nasoenteral access until hospital day (HD) 3 and had limited oral (PO) intake.

**Management Approach:**

Total fluid administration in the first 24 hours was 233 mL/kg. Despite this significant volume, the patient only tolerated conservative fluid wean. Maintenance rate of LR was achieved at hour 36 and albumin infusion was stopped at hour 74. Hypotension was minimally responsive to crystalloid bolus, requiring plasma transfusions on HD 2 and 4. On HD 4, patient developed fulminant acute respiratory distress syndrome (ARDS). He received aggressive diuresis for pulmonary edema with increasing oxygen requirements; despite this, the patient required intubation on HD 5. Parenteral nutrition was also started at this time due to enteral intolerance.

**Outcomes:**

The patient underwent a tracheostomy on HD 18 where he was weaned to minimal oxygen support with a trach mask on HD 24. The patient transitioned from TPN to enteral feeds on HD 13 and continued to have healing progression during his hospital stay.

**Lessons Learned:**

Despite providing colloid per our protocol, the patient required high fluid volumes with a prolonged wean. We suspect this is due to malnutrition related to alcohol misuse and chronic poor PO intake with emesis.

**Applicability to Practice:**

This case provides insights into the challenges of burn resuscitation in patients with malnutrition prior to injury which may be useful to other clinicians providing care to burn patients.

